# Fruit and vegetable intake in relation to gastric cancer risk: A comprehensive and updated systematic review and dose-response meta-analysis of cohort studies

**DOI:** 10.3389/fnut.2023.973171

**Published:** 2023-02-06

**Authors:** Mohammad Naemi Kermanshahi, Ehsan Safaei, Helda Tutunchi, Sina Naghshi, Sara Mobarak, Masoomeh Asadi, Omid Sadeghi

**Affiliations:** ^1^Student Research Committee, Nutrition Research Center, School of Nutrition and Food Sciences, Tabriz University of Medical Sciences, Tabriz, Iran; ^2^Nutrition Research Center, School of Nutrition and Food Sciences, Tabriz University of Medical Sciences, Tabriz, Iran; ^3^Endocrine Research Center, Tabriz University of Medical Sciences, Tabriz, Iran; ^4^Abadan Faculty of Medical Sciences, Abadan, Iran; ^5^Department of Operating Room Nursing, Abadan Faculty of Medical Sciences, Abadan, Iran; ^6^Nutrition and Food Security Research Center, Department of Community Nutrition, School of Nutrition and Food Science, Isfahan University of Medical Sciences, Isfahan, Iran; ^7^Department of Community Nutrition, School of Nutritional Sciences and Dietetics, Tehran University of Medical Sciences, Tehran, Iran

**Keywords:** fruit, gastric cancer, vegetable, citrus, meta-analysis

## Abstract

**Background:**

Since the release of previous meta-analyses, some studies on the associations between fruit and vegetable intake with gastric cancer risk have been published. Therefore, we aimed to update the previous meta-analyses on these associations by including recently published studies as well as considering the main limitations of those meta-analyses.

**Methods:**

A comprehensive search was conducted in online databases including PubMed, Scopus, ISI Web of Science, and Google Scholar to detect relevant prospective cohort studies published up to October 2021. Summary relative risks (RRs) were estimated using a random-effects model.

**Results:**

Overall, 17 articles containing 18 prospective studies with a total sample size of 1,527,995 participants, aged between 18 and 90 years, were included in the current meta-analysis. During the follow-up periods ranging between 4.5 and 21 years, 8,477 cases of gastric cancer were diagnosed. A higher intake of total fruit [RR: 0.87, 95% confidence interval (CI): 0.80 to 0.94, *I*^2^ = 0%] and total fruit and vegetable (RR: 0.75, 95% CI: 0.61 to 0.93, *I*^2^ = 55.2%) were associated with a lower risk of gastric cancer. For total vegetable intake, a significant inverse association was found among the studies that controlled their analysis for energy intake. Based on the linear dose-response analysis, each 100 g/day increase in total fruit intake (Pooled RR: 0.95, 95% CI: 0.90 to 0.99, *I*^2^ = 49%) and 200 g/day increase in total fruit and vegetable intake (RR: 0.94, 95% CI: 0.88 to 0.99, *I*^2^ = 37.6%) were associated with a 5 and 6% lower risk of gastric cancer, respectively.

**Conclusion:**

Fruit and vegetable consumption has a protective association with gastric cancer risk.

## Introduction

Although the incidence of gastric cancer is declining, it ranks as the third cause of cancer mortality (1). Previous studies have extensively assessed the association between dietary factors and gastric cancer (2, 3). Among these factors, fruits and vegetables have devoted considerable attention. These food groups contain high amounts of fiber and antioxidants that are hypothesized to protect against some cancers (4, 5). Antioxidant properties of fruits and vegetables can scavenge potentially mutagenic free radicals and induce the production of detoxification enzymes, which might counteract DNA damage caused by H. pylori (6–8). In line with the mechanisms, findings from the case-control studies published until 2007 revealed a protective association between fruit and vegetable intake and gastric cancer (9–11). However, some prospective cohort studies published since then showed opposite findings. For example, in the European Prospective Investigation into Cancer (EPIC) study, Gonzalez et al. (12) reported no significant association between vegetable intake and gastric cancer risk. Such finding was also shown in the Shanghai Women’s and Men’s Health studies (SWHS and SMHS) (13). Therefore, the judgment for the beneficial effects of fruit and vegetables has been downgraded regarding conflicting results (12–28).

In a meta-analysis in 2014, Wang et al. (29) reported that fruit intake, but not vegetables, reduced the risk of gastric cancer. However, the meta-analysis of Wu et al. (30) showed a significant inverse association between cruciferous vegetable consumption and risk of gastric cancer. Since the release of these meta-analyses, two articles containing seven prospective cohort studies have been published on the link between fruit/vegetable intake and gastric cancer (27, 28). In addition, previous meta-analyses combined risk estimates of gastric cancer mortality with those from cancer incidence made their findings misleading. Studies on cancer mortality usually do not consider alive cancer cases for calculating risk estimates. Therefore, combining these studies with those on cancer incidence attenuates the overall risk estimate obtained in a meta-analysis. Furthermore, to date, the dose-response associations of total fruit and vegetable intake and also citrus intake with gastric cancer risk have not been studied.

Given the points mentioned above, we conducted the current comprehensive and updated systematic review and dose-response meta-analysis of prospective cohort studies to review available findings on the association of total fruit, total vegetable, total fruit and vegetable, and total citrus intake with gastric cancer risk in adults.

## Methods

We used the Preferred Reporting Items for Systematic Review and Meta-Analysis (PRISMA) guidelines to report the findings of this systematic review and meta-analysis (31).

### Search strategy

A comprehensive search was conducted in the online databases including PubMed, Scopus, ISI Web of Science, and Google Scholar to detect relevant papers that assessed total fruit, total vegetable, total fruit and vegetable, and total citrus intake in relation to gastric cancer risk published to October 2021. We developed and performed the literature search (SN), and two reviewers (ES and MN) screened the titles and abstracts. The MeSH (medical subject heading terms) and non-MeSH terms used in the search strategy are presented in Supplementary Table 1. The literature search was not limited to publication time or the language of articles. After finding the relevant articles, the reference lists of them and also recent reviews were reviewed to find possible missing articles.

### Inclusion criteria

Two of the authors (ES and MN) screened the title and abstract of all publications found in the systematic search to identify studies that met our inclusion criteria. We included studies with prospective cohort design that were performed on adults (≥ 18 years) and assessed dietary intake of total fruit, total vegetable, total fruit and vegetable, or total citrus as an exposure variable and the risk of gastric cancer at any histological sites as an outcome variable. In addition, we only included the studies that reported risk estimates, including hazard ratios (HRs) and risk ratios (RRs) with 95% confidence intervals (CI) for the association between exposure and outcome. If results from one dataset were published in more than one paper, we selected the most recent one; otherwise, the one with the more complete data or with higher quality was included.

### Exclusion criteria

We did not include letters, comments, reviews, and ecological studies. Also, we excluded qualitative studies, studies that were conducted on children and adolescences, those that investigated other gastric disorders such as gastritis rather than gastric cancer, and articles that reported unadjusted risk estimates or with insufficient data (studies that did not report RR or 95% CI for the link between exposure and outcome). Moreover, we did not include those studies that assessed dietary intakes of pickled or canned vegetables, raw vegetables, green-yellow vegetables, other vegetables, apple fruits or other individual fruits rather than total fruits or vegetables, in relation to gastric cancer. One exception is a study (21) in which fresh vegetables accounts for a very large proportion of total vegetables, and in this case reported risk estimate was included in the meta-analysis.

### Data extraction

Two investigators independently extracted required data from each paper (MN and ES). Since the main risk estimates in the current meta-analysis were RRs, or HRs along with 95% CIs, we extracted them from included articles. We extracted the risk estimates in the fully adjusted model if an article contained crude and multivariable-adjusted models. In addition to risk estimates, the following information was extracted from each article: the first author name, publication year, sample size, number of cancer cases, demographic characteristics of participants (age range or mean age, gender), study location, duration of follow-up, methods used to assess dietary intake and gastric cancer, and confounding variables taking into account in the statistical analysis.

### Quality assessment

The quality of included studies was determined using the Newcastle Ottawa Scale, designed for non-randomized studies (32). Based on this scale, an article can get a maximum score of 9 given the following parameters: 4 points for selection of participants, 2 points for comparability, and 3 points for the assessment of outcomes. We considered studies with 0–3, 4–6, and 7–9 points to represent low, medium, and high quality studies, respectively.

### Statistical methods

We included the risk estimates (including HRs and RRs) and 95% CIs of gastric cancer for the comparison between the highest and lowest intakes of fruit/vegetable intake into the meta-analysis. We first calculated the natural log form (and its standard error) of these RRs and then we combined them using a random-effects model to calculate the overall RR of gastric cancer for the comparison between the highest and lowest intakes of fruits/vegetables. Compared to a fixed-effects model, a random-effects model can take between-study heterogeneity into account (33). For studies that provided results by sex or other subgroups, we first pooled these estimates using a fixed-effects model and included the pooled value in the main analysis. In addition, we calculated both Q-statistic and *I*^2^ values as indicators of heterogeneity. We considered the *I*^2^ values of < 25%, 25–50%, 50–75%, and > 75% as low, moderate, high, and very high between-study heterogeneity, respectively (34). We performed subgroup analyses based on some important variables such as study location (USA vs. non-USA), follow-up duration (≥ 10 vs. < 10 years), participants’ gender, dietary assessment methods (FFQ vs. dietary recall), and adjustments for body mass index (BMI) and dietary energy intake (adjusted vs. non-adjusted) to detect possible sources of heterogeneity. Publication bias was examined using Egger’s linear regression test (35). In the case of substantial publication bias, the trim-and-fill method was used to detect the effect of probable missing studies on the overall RR (36). To assess the dependency of overall RR on one study, the sensitivity analysis was conducted using a random-effects model in which each study was excluded to examine the influence of that study on the overall estimate.

We used the generalized least squares trend estimation method described by Greenland and Longnecker (37) and Orsini et al. (38) for the linear dose-response analysis. First, study-specific slopes were estimated, and then, these slopes were combined to obtain an overall average slope. Study-specific slopes were combined using a random-effects model. In this method, the distribution of gastric cancer cases, the total number of participants, and the RRs with the variance estimates for ≥ 3 quantitative categories of exposure were required. We assigned the median or mean amount of fruit/vegetable intake in each category to the corresponding RR for each study. For studies that reported the intake as ranges, we estimated the midpoint in each category by calculating the mean of the lower and upper bound. When the highest category was open-ended, the length of the open-ended interval was assumed to be the same as that of the adjacent interval. Consistent with previous meta-analyses, we used 80 g as a serving size for fruit and vegetable intake (39, 40). We also examined a possible non-linear dose-response relationship using restricted cubic splines with 3 knots at percentiles of 10, 50,and 90% of the distribution (41). The correlation within each set of provided risk estimates was taken into account and the study-specific estimates were combined by using a one-stage linear mixed-effects meta-analysis (42). This method estimates the study-specific slopes and combines them to obtain an overall average slope in a single stage, and is a more precise, flexible, and efficient method than the traditional two-stage method (42). The significance for non-linearity was calculated by null hypothesis testing, in which the coefficient of the second spline was considered equal to zero. Statistical analyses were conducted using STATA version 14.0. *P* < 0.05 was considered as statistically significant for all tests, including Cochran’s *Q* test.

## Results

### Findings from the systematic search

**FIGURE 1:**
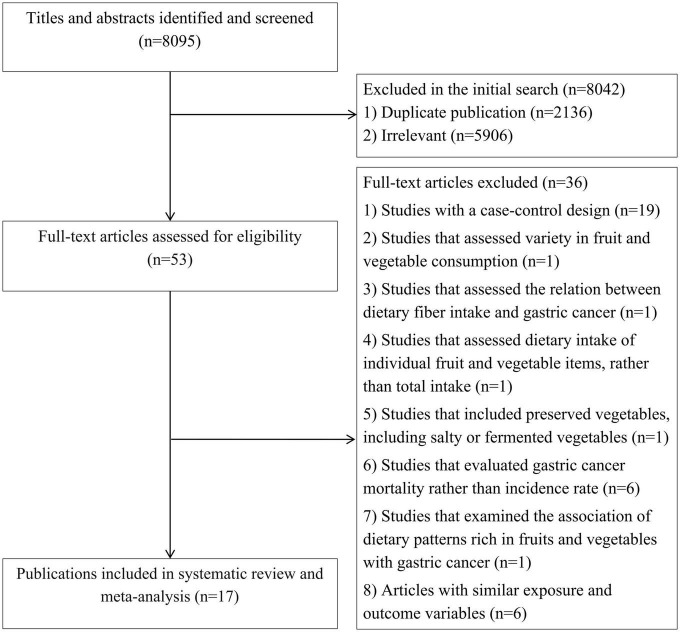
Flow diagram of study selection.

In our initial search in the online databases, we found 8,095 articles that 2136 of them were duplicates and therefore were excluded. Of the remaining articles, 5,906 were also excluded because they were unrelated to our subject based on assessing their title and abstract. In total, 53 papers remained for full-text assessment, of them, 19 were excluded because of case-control design. Also, Jeurnink et al. (43) study was excluded because they assessed variety in fruit and vegetable consumption rather than their intakes. The study of Botterweck et al. (44) that assessed the relation between dietary fiber intake and gastric cancer was excluded as well. In addition, we excluded another study that assessed dietary intake of individual fruit and vegetable items, rather than total intake, in relation to gastric cancer (45). The study of Ji et al. (46) was excluded because they included preserved vegetables, including salty or fermented vegetables in their analysis. Some studies evaluated gastric cancer mortality rather than incidence rate and therefore were excluded (47–52). The study of Masaki et al. (53) was not included because they examined the association of dietary patterns rich in fruits and vegetables with gastric cancer. Moreover, we found duplicate papers on datasets of European Prospective Investigation into Cancer and Nutrition (EPIC) study (12, 54, 55), Netherlands Cohort Study (25, 56), Japan Collaborative Cohort Study (27, 57), Japan Public Health Center-based prospective Study (27, 58), and Linxian General Population Trial Cohort (21, 59). Among these articles, we retained the articles with more complete findings or with higher quality (12, 21, 25, 27) and excluded the six duplicate papers. In addition, we found one article that was a pooled analysis on five cohort studies, including Shanghai Women’s Health Study (SMHS), Shanghai Women’s Health Study (SWHS), Japan Public Health Center-based Prospective Study I and II (JPHC I and II), and Korean Cancer Prevention Study (KCPS) from which only the KCPS was not duplicate and other datasets were present in the articles included in the current meta-analysis. To avoid double-counting data and missing any datasets, we did not include the article in the main meta-analysis and we just did a sensitivity analysis for that. In total, 17 articles containing 18 prospective studies were included in the current systematic review and meta-analysis, of them, 11 papers assessed total vegetable intake (12–14, 20–23, 25–28), 13 articles total fruit intake (12–14, 16, 18, 20–23, 25–28), 6 publications total intake of fruits and vegetables (12, 17, 19, 22, 23, 27), and 5 publications citrus intake in relation to gastric cancer risk (12, 13, 23–25). Flow diagram of study selection is presented in Figure 1.

### Characteristics of included studies

Details on the characteristics of prospective studies included in the current meta-analysis are shown in Supplementary Table 2. The 17 articles that were published between 1990 and 2017 contained prospective cohort studies with a follow-up period ranging between 4.5 and 21 years. These studies contained a total sample size of 1,522,911 participants, aged between 18 and 90 years, and 8,477 cases of gastric cancer. The number of participants varied between 742 and 490,802 individuals in these studies. All studies were conducted on both genders except Nomura et al. (15) and Chyou et al. (14) that were done on males only. Of the 17 papers, ten articles described studies that were conducted in Western countries, including the USA (14, 15, 18, 23) and European countries (12, 17, 19, 20, 22, 25) and the remaining seven publications described studies that were carried out in Asian countries (13, 16, 21, 24, 26–28). All studies except three used food frequency questionnaires for dietary assessment. Of the three studies, one used food recall (14), Galanis et al. applied weekly frequency of foods (18), and another study used a researcher-designed questionnaire to assess dietary intakes (19). Fifteen articles considered gastric cancer incidence as the outcome of interest, while in two publications (13, 28), the incidence of non-cardia gastric cancer was considered as the outcome variable. In all included studies, gastric cancer was diagnosed using medical data obtained from medical records and cancer registries. In the most included publications, some important confounders including age (*n* = 17), BMI (*n* = 5), smoking (*n* = 14), alcohol consumption (*n* = 8), physical activity (*n* = 3), and energy intake (*n* = 8) were adjusted in the analysis of fruit and vegetable intake with gastric cancer risk. Based on the NOS, all included studies except two (17, 26) were considered high-quality studies (Supplementary Table 3).

### Findings from the systematic review

Among the 11 articles on the association between total vegetable intake and gastric cancer risk, only one study showed a significant inverse association (22) and others reported a non-significant association. Also, two article among the 13 papers on total fruit intake revealed a significant inverse association with gastric cancer (18, 28), while others showed a non-significant association. Of the six papers on the link between total intake of fruits and vegetables, three studies showed a significant inverse association with risk of gastric cancer (19, 22, 27) and others indicated no significant association. Also, one article (25), out of four papers, showed a significant inverse association between citrus intake and gastric cancer risk.

### Findings from the meta-analysis

All studies except Wang et al. article (28) were included in the main meta-analysis. Since the Wang et al. article that contained a pooled analysis of five prospective cohort studies had a high overlap with other included studies, we performed the main analyses without this article to avoid double-counting data. However, a sensitivity analysis was done for that article.

### Total vegetable intake and gastric cancer

**FIGURE 2:**
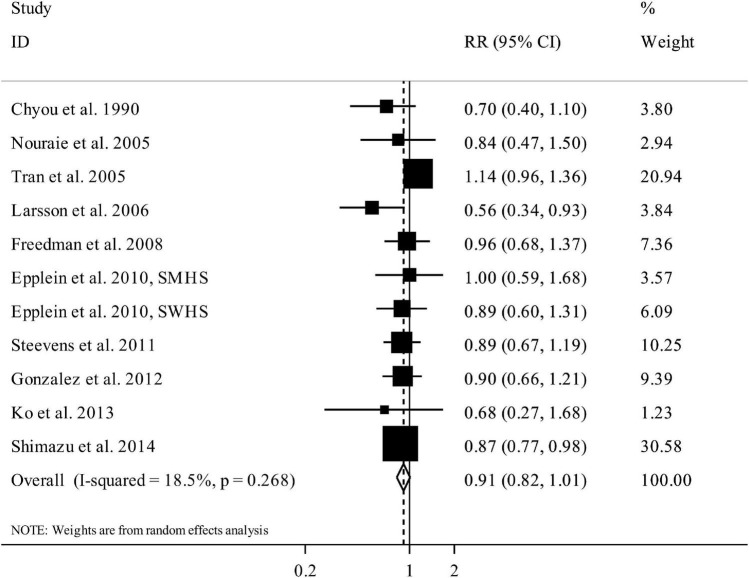
Forest plot for the association between total vegetable intake and risk of gastric cancer in adults aged > 18 years, expressed as the comparison between the highest and lowest categories of total vegetable intake. Horizontal lines represent 95% CIs. Diamonds represent the pooled estimates from the random-effects analysis. RR, relative risk; CI, confidence interval.

In total, 10 papers (containing 15 studies) with a total sample size of 1,445,175 participants and 7,075 cases of gastric cancer assessed the link between vegetable intake and gastric cancer (12–14, 20–23, 25–27). The summary RR for gastric cancer risk, comparing the highest and lowest categories of total vegetable intake, was 0.91 (95% CI: 0.82 to 1.01, *I*^2^ = 18.5%, *P*_heterogeneity_ = 0.26), indicating no significant association between total vegetable intake and gastric cancer (Figure 2). However, the summary RR depended on the Tran et al. article so that by excluding the RR of this publication, the overall RR became significant (RR:0.86, 95% CI: 0.79 to 0.95, *I*^2^ = 0, *P*_heterogeneity_ = 0.87).

**FIGURE 3:**
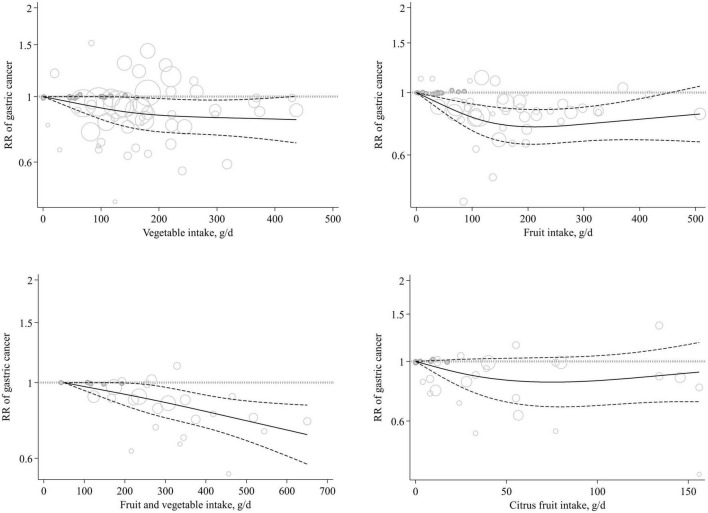
Non-linear dose-response association of total vegetable, total fruit, citrus fruit, and total fruit and vegetable intakes (based on g/day) with risk of gastric cancer in adults aged ≥ 18 years. The solid lines indicate the spline model. The dashed lines present the 95% CI. RR, relative risk; CI, confidence interval.

All studies in this section had complete data for the dose-response analysis. In the linear dose-response analysis, we found no significant association between total vegetable intake and gastric cancer risk based on a 100 g/day increase in vegetable intake (Pooled RR: 0.96, 95% CI: 0.92 to 1.00, *I*^2^ = 37.4%, *P*_heterogeneity_ = 0.10) (Supplementary Figure 1). Also, we found no evidence of non-linear association between total vegetable intake and gastric cancer (*P* non-linearity = 0.26) (Figure 3).

### Total fruit intake and gastric cancer

**FIGURE 4:**
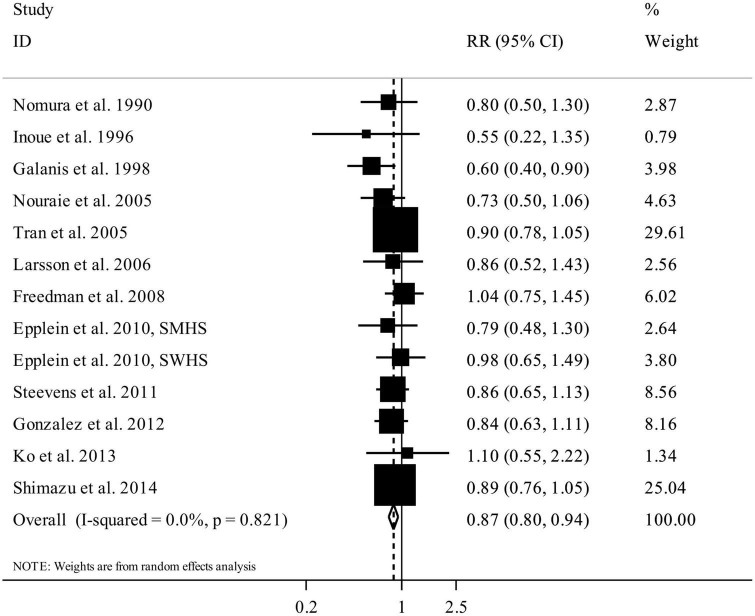
Forest plot for the association between total fruit intake and risk of gastric cancer in adults aged > 18 years, expressed as the comparison between the highest and lowest categories of total fruit intake. Horizontal lines represent 95% CIs. Diamonds represent the pooled estimates from the random-effects analysis. RR, relative risk; CI, confidence interval.

We included 12 articles (16 studies) with a total sample size of 1,465,515 participants and 7,330 cases of gastric cancer in this section (12, 13, 15, 16, 18, 20–23, 25–27). The summary RR for the risk of gastric cancer, comparing the highest versus lowest intake of total fruits, was 0.87 (95% CI: 0.80 to 0.94, *I*^2^ = 0%, *P*_heterogeneity_ = 0.82), illustrating a significant inverse association between total fruit intake and gastric cancer (Figure 4).

Of the 12 articles, 9 had complete data for inclusion in the dose-response analysis (12, 13, 15, 20, 22, 23, 25–27). Linear dose-response analysis showed that each 100 g/day increase in total fruit intake was associated with a 5% lower risk of gastric cancer (Pooled RR: 0.95, 95% CI: 0.90 to 0.99, *I*^2^ = 49%, *P*_heterogeneity_ = 0.03) (Supplementary Figure 2). Also, we found a non-linear association between total fruit intake and gastric cancer (*P* non-linearity = 0.004), with a significant reduction in risk from no intake up to 200 g/day and there was no further reduction in risk above the 200 g/day (Figure 3).

### Total fruit and vegetable intake and gastric cancer

**FIGURE 5:**
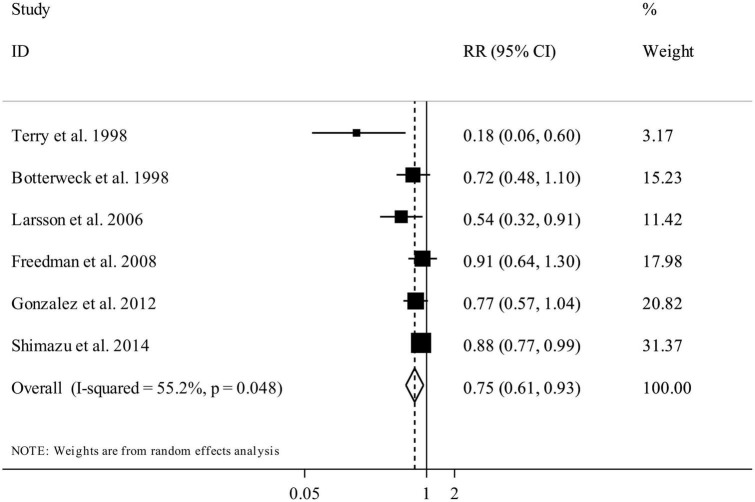
Forest plot for the association between total fruit and vegetable intake and risk of gastric cancer in adults aged > 18 years, expressed as the comparison between the highest and lowest categories of total fruit and vegetable intake. Horizontal lines represent 95% CIs. Diamonds represent the pooled estimates from the random-effects analysis. RR, relative risk; CI, confidence interval.

Six publications (nine studies) entered total intake of fruits and vegetables, rather than total fruits or total vegetables, in their analysis (12, 17, 19, 22, 23, 27). These studies included 1,256,299 individuals and 4,591 cases of gastric cancer. Combining data from these studies, comparing the highest versus lowest categories of total fruit and vegetable intake showed a summary RR of 0.75 (95% CI: 0.61 to 0.93, *I*^2^ = 55.2%, *P*_heterogeneity_ = 0.04), indicating a significant inverse association in this regard (Figure 5).

Eight studies, out of the nine studies, had complete data for performing the dose-response analysis. Based on the linear dose-response analysis, we found a significant inverse association between a 200 g/day increase in total fruit and vegetable intake and gastric cancer risk (Pooled RR: 0.94, 95% CI: 0.88 to 0.99, *I*^2^ = 37.6%, *P*_heterogeneity_ = 0.17) (Supplementary Figure 3). In the non-linear dose-response analysis, we found no evidence of non-linearity (*P* non-linearity = 0.82) (Figure 3).

### Citrus intake and gastric cancer

**FIGURE 6:**
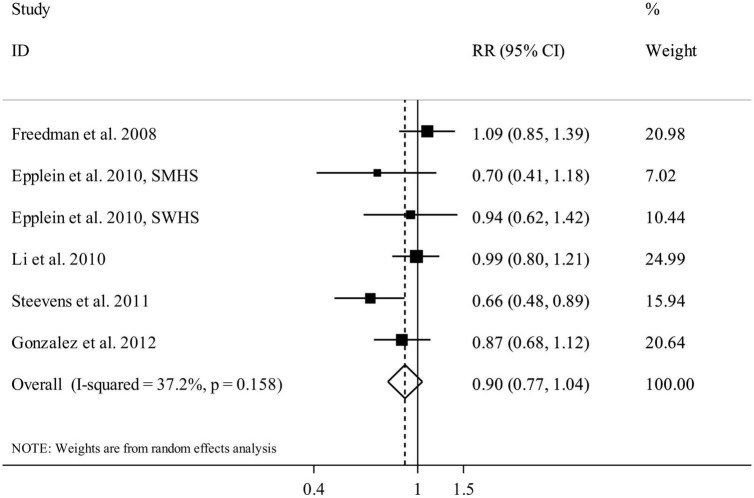
Forest plot for the association between citrus fruit intake and risk of gastric cancer in adults aged > 18 years, expressed as the comparison between the highest and lowest categories of citrus fruit intake. Horizontal lines represent 95% CIs. Diamonds represent the pooled estimates from the random-effects analysis. RR, relative risk; CI, confidence interval.

Five articles (six studies) containing 1,147,546 participants and 2,837 gastric cancer cases were included in the analysis of citrus intake and gastric cancer (12, 13, 23–25). The summary RR for gastric cancer, comparing the highest and lowest categories of citrus intake, was 0.90 (95% CI: 0.77 to 1.04, *I*^2^ = 37.2%, *P*_heterogeneity_ = 0.15) that indicated a non-significant association (Figure 6). In the dose-response analysis, we found no significant linear association between citrus intake and gastric cancer (Pooled RR: 0.98, 95% CI: 0.94 to 1.02, *I*^2^ = 0%, *P*_heterogeneity_ = 0.58) (Supplementary Figure 4). In addition, the test for non-linearity was not significant (*P* non-linearity = 0.17) (Figure 3).

### Subgroup analysis, sensitivity analyses, and publication bias

Findings from the subgroup analyses are shown in Supplementary Table 4. In terms of total vegetable intake, we found a significant inverse association among the studies that controlled their analysis for energy intake and those that examined gastric non-cardia cancer risk. Regarding total fruit intake, we found a significant inverse association among the studies that conducted in non-USA countries, those that examined gastric non-cardia cancer risk, studies with a follow-up period ≥ 10 years, studies that conducted on both genders, studies that applied FFQ for dietary assessment, studies that controlled for energy intake, and those that did not adjust for BMI and energy in their analysis. For total fruit and vegetable intake and gastric cancer, we found a significant inverse association in non-USA studies, those with a follow-up period < 10 years, studies that applied FFQ for dietary assessment, studies controlled for energy intake, and those that did not adjust for BMI in their analysis. In terms of citrus fruit intake, a significant inverse association was observed in non-USA studies, studies that examined gastric cardia cancer risk, and those that did not adjust for BMI in their analysis.

Based on sensitivity analysis, our findings on the link between total vegetable intake and gastric cancer depended on Tran et al. study (21) so that after excluding that study, the non-significant inverse association between total vegetable intake and gastric cancer became significant (Pooled RR:0.86, 95% CI: 0.79 to 0.95, *I*^2^ = 0, *P*_heterogeneity_ = 0.87). Also, excluding the study of Freedman et al. (Pooled RR: 0.85, 95% CI: 0.73 to 0.99, *I*^2^ = 25.3%, *P*_heterogeneity_ = 0.25) resulted in a significant association between citrus intake and gastric cancer. Sensitivity analyses for the Wang et al. article (28) showed that including the findings of this paper in the analyses of total vegetable intake (Pooled RR: 0.92, 95% CI: 0.84 to 1.00, *I*^2^ = 10.5%, *P*_heterogeneity_ = 0.34) and total fruit intake (Pooled RR: 0.85, 95% CI: 0.79 to 0.92, *I*^2^ = 0%, *P*_heterogeneity_ = 0.62) did not change the overall results obtained for these exposures. Of note, this article did not present data on citrus intake and total vegetable and fruit intake. In terms of publication bias, Egger’s linear regression test showed no substantial publication bias in all associations except for total fruit and vegetable intake and gastric cancer, in which there was evidence of publication bias. Nevertheless, the application of the trim-and-fill method did not change the average effect size, further suggesting that results were not affected by publication bias.

## Discussion

In the current meta-analysis, we found that a higher intake of total fruit and total fruit and vegetable was associated with a 13 and 25% lower risk of gastric cancer, respectively. Although the overall association was not significant for total vegetable intake, we found a significant inverse association among the studies that controlled their analysis for energy intake. Moreover, in the linear dose-response analysis, each 100 g/day increase in total fruit intake and 200 g/day increase in total fruit and vegetable intake were associated with a 5 and 6% lower risk of gastric cancer, respectively. Such linear association was not seen for total vegetable intake and also citrus intake.

Gastric cancer is a major public health concern worldwide due to its frequency, limited therapies, and poor prognosis (1). Previous studies examined the contribution of dietary factors for cancer prevention, particularly gastric cancer (60–64). Fruits and vegetables are one of the main portions of a diet. However, the influence of these food groups on stomach is still unknown. Epidemiological studies illustrated inconsistent findings on the association of fruit and vegetable intake with gastric cancer. To the best of our knowledge, this study is among the first comprehensive meta-analyses to summarize prior publications on the association of fruit and vegetable intake with gastric cancer risk. Compared with previous meta-analysis (29), we included additional studies with a larger number of gastric cancer cases and participants. Moreover, earlier meta-analysis (29) of gastric cancer risk had several limitations that make their findings misleading.

In the current meta-analysis, we found that total fruit intake had a protective association with gastric cancer. This finding was similar to the previous meta-analysis conducted by Wang et al. (29) in which total fruit intake was associated with a reduced risk of gastric cancer. A meta-analysis of case-control studies investigating the association between fruit intake and gastric cancer showed such a significant inverse association (39). In total, it seems that foods with high antioxidant capacity suppress the progression of atrophic gastritis to carcinoma (39). Fruits are the main source of vitamin C and polyphenols that both inhibit the production of carcinogenic *N*-nitroso compounds in humans (65). In the current study, there was evidence of a non-linear association between total fruit intake and risk of gastric cancer, with a significant reduction in risk from no intake up to 200 g/day and there was no further reduction in risk above the 200 g/day. Therefore, based on a public health perspective, targeting populations with a low intake of fruit might be most effective for preventing gastric cancer incidence. In the current study, we failed to find any significant association between citrus fruit intake and gastric cancer risk. This non-significant association might be explained by a limited number of included studies in this relation. More studies are needed to clarify the association of citrus fruit intake with gastric cancer risk. We found a difference in the association between fruit intake and gastric cancer risk according to the length of follow-up, with a significant association for those studies that had 10 years or more of follow-up compared with those that had less than 10 years of follow up. This might be explained by higher sample sizes or gastric cancer events in studies that had 10 years or more of follow-up duration, which provided increased statistical power to detect significant association. A significant inverse association between fruit intake and gastric cancer was also seen among the non-USA population, while this association was not significant in the USA population. A possible explanation for this regional difference is the relatively higher intake of fruits among non-USA populations. Moreover, differences in types, preparation methods, consumption habits of fruits among USA and non-USA populations may play a role. Additionally, power may have been low to detect a significant association among USA population because of a low number of the included studies. The differential associations observed between fruit or vegetable intake with cardia and non-cardia gastric cancer might be explained by different etiology of these two types of gastric cancer (66). These findings imply that the potential beneficial effects of fruit and vegetable may not be uniform across gastric cancer subsites.

In the case of total vegetable intake, we found no significant association with gastric cancer risk in the overall analysis. However, the results depended on Tran et al. study (21) so that by excluding the RR of this study, the non-significant inverse association between total vegetable intake and gastric cancer became significant. It must be kept in mind that the study of Tran et al. did not control their analysis for key confounding variables such as BMI, smoking, alcohol and energy intake. Lack of controlling for such confounders might affect the independent association of vegetable intake and risk of gastric cancer. Moreover, based on the non-linear dose-response analysis, we found a significant inverse association between vegetable intake and gastric cancer risk in the dietary intakes from 130 to 400 g/day. It seems that findings from the dose-response meta-analyses are more reliable than those from the highest versus lowest intake comparison in which estimates might encounter misclassification bias because of the different ranges of the highest and lowest categories of vegetable intakes among different studies.

When we confined the analysis to studies that controlled for energy intake in their analysis, a significant inverse association was seen between total vegetable intake and gastric cancer. In the meta-analysis of Wang et al. (29) no significant association between total vegetable intake and gastric cancer was reported either by considering total studies or those with statistical adjustment for dietary energy intake. This inconsistency in the subgroup analysis might be explained by different criteria for including eligible studies. In the Wang et al. (29) study, authors included studies on gastric cancer mortality in the meta-analysis of gastric cancer risk, making their findings misleading. Studies on cancer mortality usually do not consider alive cancer cases for calculating risk estimates and this makes bias when we include the findings of these studies in the meta-analysis of cancer incidence. Unlike Wang et al. (29) study, we included studies on gastric cancer incidence in the meta-analysis only.

It should be kept in mind that different types of vegetables may have different associations with the risk of gastric cancer. For instance, in a meta-analysis, Wu et al. (30) reported that cruciferous vegetable consumption was inversely associated with the risk of gastric cancer and non-cardia gastric cancer, while another meta-analysis on pickled vegetables showed that each 40 g/day increase in intake of this food group was associated with a 15% higher risk of gastric cancer (67). Therefore, types of vegetables play an important role in determining the overall association of total vegetable intake with gastric cancer risk. In addition, different cooking and processing methods used for preparing vegetables in different cultures may affect this association.

Vegetables are a rich source of fiber and antioxidants that induce cancer-preventive effects. The meta-analysis of Zhang et al. (68) indicated that a higher intake of dietary fiber was associated with a reduced risk of gastric cancer. Dietary fiber reduces the concentrations of N-nitroso compounds, which are potentially carcinogenic in humans (69). Vegetables are rich sources of ferulic acid and p-coumaric acid, which could delay the progression of the cell cycle and produce anticancer effects (70). In contrast, some types of vegetables, such as pickled or canned vegetables, are exogenous sources of sodium nitrates and nitrites, which by a reaction with amino acids in the stomach can produce N-nitro compounds (71). In total, different types of vegetables might have different effects on health outcomes. Future studies should determine the association between individual vegetables and gastric cancer.

Since small studies with non-significant results or unattractive findings tend not to be published, the possibility of publication bias is unavoidable in meta-analyses. This bias is particularly common among observational studies (72). In the current meta-analysis, we found significant publication bias for the relation between total fruit and vegetable intake and gastric cancer. However, filling the possible missing studies using the application of the trim-and-fill method did not change our findings on the association. It means that publication bias did not affect our findings on the link between total fruit and vegetable intake and gastric cancer.

The major strength of this meta-analysis was the prospective design of included studies. Prospective studies have the highest quality among observational studies. Also, the linear and non-linear dose-response analysis provided the most compelling evidence for quantitative evaluation of associations and enabled us to determine the strength and shape of the dose-response relationships. In addition, between-study heterogeneity was low in most associations evaluated in the current meta-analysis. Our findings should be interpreted by considering several limitations. First, since the studies included in the present meta-analysis were observational in nature, causality cannot be established. Second, the role of residual confounders from unmeasured behavioral and biological factors or errors in the measurement of covariates cannot be entirely excluded because of the observational design of included studies. Third, measurement errors are inevitable in estimation of fruit and vegetable intakes. Misclassification due to measurement errors could result in underestimating the associations of fruit and vegetable intake with the risk of gastric cancer. Fourth, as usually the case in cohort studies, most included studies had estimated dietary intakes based on a single measurement at study baseline, and changes in the diet throughout the follow-up were not considered. In addition, regional differences in fruit and vegetable intake may have been an issue in this meta-analysis that might affect the highest and lowest categories of exposures and the results obtained from the comparison of these categories. However, we performed a subgroup analysis accordingly to control these differences. We also conducted the dose-response analysis as another strategy to control these differences and the overlap between the ranges of fruit and vegetable intake among different studies.

In conclusion, a greater intake of total fruits and total fruits and vegetables were associated with a 13 and 25% lower risk of gastric cancer, respectively. Also, when we combined the studies that excluded the confounding effect of energy intake from their analysis, such inverse association was seen for total vegetable intake. Also, each 100 g/day increase in total fruit intake and each 200 g/day increase in total fruit and vegetable intake were associated with a 5 and 6% lower risk of gastric cancer. Further studies should examine the associations of individual fruits and vegetables with gastric cancer risk.

## Data availability statement

The original contributions presented in this study are included in the article/Supplementary material, further inquiries can be directed to the corresponding authors.

## Author contributions

MN and ES contributed to the literature search and data extraction. SN and OS contributed to the data analysis. OS drafted the manuscript which was critically revised for important intellectual content by ES, HT, MA, MN, OS, SM, and SN. HT and ES contributed to the manuscript drafting and data analysis. SM and MA obtained funding and contributed to the manuscript editing. OS supervised the study. All authors have read and approved the final manuscript.
