# Supplementary material for: Fruit and vegetable intake in relation to gastric cancer risk: A comprehensive and updated systematic review and dose-response meta-analysis of cohort studies
Source: Front Nutr. 2023 Feb 6;10:973171. doi: 10.3389/fnut.2023.973171 (PMC9939448; doi:10.3389/fnut.2023.973171)
Supplement: Supplementary file 1 [file Data_Sheet_1.docx]

**Supplemental Table 1**: Medical subject headings (MeSH) and non-MeSH terms used to search relevant publications on the relation between fruit and vegetable intake and risk of gastric cancer^1^

| **Concept 1** | (Fruit OR vegetable OR plant OR vegetablesOR Citrus OR diet OR consumption) |
| --- | --- |
| **Concept 2** | (“Stomach Neoplasms” OR “Stomach Neoplasm” OR “Gastric Neoplasms” OR “Stomach Cancer” OR “Gastric Cancers” OR “Gastric Cancer” OR “Gastric Malignancy” OR “Gastric Tumor” OR “Gastric Carcinoma”) |

^1^The combination of terms as mentioned above was used to search online databases: ("concept 1" AND "concept 2")

**Supplemental Table 2**: Characteristics of included studies on the association between fruit and vegetable intake and risk of gastric cancer in adults aged ≥18 y

| Author | Country | Age, year | Sample size, *n* | Follow up, year | Cases, *n* | Exposure | Exposure assessment | Outcome | Outcome assessment | Median/ cutoff point | RR (95% CI) | Adjustment |
| --- | --- | --- | --- | --- | --- | --- | --- | --- | --- | --- | --- | --- |
| Steevens et al. 2011 | Netherlands | 55-69 | F/M: 4,651 | 16.3 | 148 | Vegetable | FFQ | GCC | Cancer registry | 104 g/d  146  181  222  297 | 1  0.63 (0.37-1.06)  0.64 (0.37-1.09)  0.87 (0.53-1.45)  0.87 (0.50-1.52) | Age, sex, cigarette smoking, alcohol consumption, consumption of red meat, consumption of fish |
|  |  |  |  |  | 443 |  |  | GNCC |  | 104 g/d  146  181  222  297 | 1  0.79 (0.58-1.08)  0.88 (0.65-1.20)  0.80 (0.58-1.10)  0.90 (0.64-1.26) |  |
|  |  |  |  |  | 156 | Fruit |  | GCC |  | 43 g/d  107  155  215  326 | 1  0.63 (0.38–1.04)  0.75 (0.46–1.21)  0.86 (0.53–1.40)  0.85 (0.50–1.42) |  |
|  |  |  |  |  | 460 |  |  | GNCC |  | 43 g/d  107  155  215  326 | 1  0.84 (0.62–1.13)  0.95 (0.70–1.28)  0.83 (0.61–1.13)  0.86 (0.62–1.18) |  |
|  |  |  |  |  | 156 | Citrus fruit |  | GCC |  | 0 g/d  8  33  77  156 | 1  0.76 (0.47-1.22)  0.54 (0.32-0.92)  0.55 (0.32-0.94)  0.38 (0.21-0.69) |  |
|  |  |  |  |  | 460 |  |  | GNCC |  | 0 g/d  8  33  77  156 | 1  0.86 (0.61-1.21)  0.89 (0.62-1.27)  0.99 (0.70-1.40)  0.80 (0.56-1.15) |  |
| Shimazu et al. 2014 | japan | 40-79 | M: 87,771 | 11 | 2995 | Vegetable | FFQ | GC | Cancer registry | 43 g/d  69  96  128  165 | 1  0.95 (0.82-1.10)  0.96 (0.83-1.10)  0.94 (0.81-1.08)  0.89 (0.77-1.03) | Age, location within the study area, smoking status, sodium intake, and total energy intake |
|  |  |  | F: 103,461 |  |  |  |  |  |  | 56 g/d  82  109  140  175 | 1  0.76 (0.61-0.94)  0.82 (0.65-1.04)  0.92 (0.75-1.13)  0.83 (0.67-1.03) |  |
|  |  |  | M: 87,771 |  |  | Fruit |  |  |  | 14 g/d  42  71  112  160 | 1  0.89 (0.72-1.11)  0.89 (0.74-1.07)  0.83 (0.72-0.97)  0.92 (0.76-1.11) |  |
|  |  |  | F: 103,461 |  |  |  |  |  |  | 43 g/d  68  106  148  194 | 1  0.85 (0.69-1.04)  0.81 (0.66-0.99)  0.68 (0.54-0.86)  0.82 (0.59-1.12) |  |
|  |  |  | M: 87,771 |  |  | Total vegetable and fruit |  |  |  | 49 g/d  123  173  235  307 | 1  0.91 (0.76-1.11)  0.97 (0.84-1.12)  0.91 (0.79-1.05)  0.87 (0.75-1.01) |  |
|  |  |  | F: 103,461 |  |  |  |  |  |  | 111 g/d  167  221  281  349 | 1  0.90 (0.69-1.17)  0.89 (0.72-1.10)  0.84 (0.68-1.05)  0.89 (0.72-1.12) |  |
| Nouraie et al. 2005 | Finland | 50-69 | F/M: 27,110 | 12 | 57 | Vegetable | FFQ | GCC | Cancer registry | <66 g/d  67-100  101-147  >148 | 1  1.52 (0.74-3.10)  0.44 (0.16-1.22)  0.81 (0.27-2.48) | Age, total years of smoking, education, and dietary nitrate |
|  |  |  |  |  | 163 |  |  | GNCC |  | <66 g/d  67-100  101-147  >148 | 1  0.94 (0.60-1.45)  0.85 (0.51-1.40)  0.85 (0.43-1.68) |  |
|  |  |  |  |  | 57 | Fruit |  | GCC |  | <61 g/d  62-107  108-166  >167 | 1  0.93 (0.44-1.97)  0.84 (0.39-1.83)  1.00 (0.47-2.18) |  |
|  |  |  |  |  | 163 |  |  | GNCC |  | <61 g/d  62-107  108-166  >167 | 1  0.41 (0.26-0.65)  0.50 (0.32-0.78)  0.66 (0.43-1.02) |  |
| Larsson et al. 2006 | Sweden | 45-83 | F/M: 82,002 | 7.2 | 139 | Vegetable | FFQ | GC | Cancer registry | <1 Servings/d  1-1.4  1.5-2.4  ≥2.5 | 1  0.66 (0.36-1.21)  0.67 (0.41-1.11)  0.57 (0.34-0.93) | Age, sex, education, smoking status and pack-years of smoking, diabetes, and intakes of total energy, alcohol, and processed meat |
|  |  |  |  |  |  | Fruit |  |  |  | <1 Servings/d  1-1.4  1.5-2.4  ≥2.5 | 1  0.93 (0.58-1.49)  0.86 (0.55-1.36)  0.86 (0.52-1.43) |  |
|  |  |  |  |  |  | Total vegetable and fruit |  |  |  | <2 Servings/d  2-3.4  3.5-4.9  ≥5 | 1  0.63 (0.38-1.02)  0.66 (0.39-1.10)  0.54 (0.32-0.91) |  |
| Gonzalez et al. 2012 | 10 European countries | 35-70 | F/M: 477,312 | 11 | 683 | Vegetable | FFQ | GC | Cancer registry | 62.5 g/d  117.5  169  244  437 | 1  0.99 (0.79–1.24)  0.94 (0.74–1.20)  0.79 (0.60–1.03)  0.90 (0.66–1.21) | Age, sex, BMI, educational  level, alcohol intake, smoking, physical activity, energy intake and consumption of red and processed meat |
|  |  |  |  |  |  | Fruit |  |  |  | 48.1 g/d  117  185  277  508 | 1  1.13 (0.90–1.42)  0.93 (0.72–1.19)  0.85 (0.65–1.10)  0.84 (0.63–1.11) |  |
|  |  |  |  |  |  | Citrus fruit |  |  |  | 1.75 g/d  11  28  56.5  145.5 | 1  0.78 (0.62–0.99)  0.84 (0.67–1.07)  0.63 (0.49–0.82)  0.87 (0.68–1.12) |  |
|  |  |  |  |  |  | Total vegetable and fruit |  |  |  | 149 g/d  266  375  517  650 | 1  1.02 (0.82–1.28)  0.78 (0.61–1.01)  0.79 (0.61–1.03)  0.77 (0.57–1.04) |  |
| Epplein et al.2010 | China | 40–74 | M: 59,247 | 3.6 | 132 | Vegetable | FFQ | GNCC | Cancer registry | ≤212.9 g/d  212.9-307.2 307.2-429.3  >429.3 | 1  1.13 (0.70-1.82)  0.99 (0.60-1.63)  1.00 (0.59-1.68) | Age, education, smoking, and total energy intake |
|  |  |  |  |  |  | Fruit |  |  |  | ≤56.5 g/d  56.5-128.3  128.3-215.7  >215.7 | 1  0.88 (0.56-1.39)  0.66 (0.40-1.11)  0.79 (0.48-1.30) |  |
|  |  |  |  |  |  | Citrus fruit |  |  |  | ≤1.6 g/d  1.6-6.3  6.3-18.0  >18.0 | 1  0.84 (0.52-1.36)  1.02 (0.65-1.62)  0.70 (0.41-1.18) |  |
|  |  |  | F: 73,064 | 9.2 | 206 | Vegetable |  |  |  | ≤179.5 g/d  179.5-261.3 261.3-373.7 >373.7 | 1  0.69 (0.47-1.01)  0.59 (0.39-0.90)  0.89 (0.60-1.31) |  |
|  |  |  |  |  |  | Fruit |  |  |  | ≤134.2 g/d  134.2-238.3  238.3-357.8  >357.8 | 1  0.88 (0.60-1.29)  1.07 (0.73-1.57)  0.98 (0.65-1.49) |  |
|  |  |  |  |  |  | Citrus fruit |  |  |  | ≤6.1 g/d  6.1-17.7  17.7-31.9  >31.9 | 1  1.00 (0.68-1.46)  1.05 (0.71-1.53)  0.94 (0.62-1.42) |  |
| Freedman et al. 2008 | US | NR | M/F: 490802 | 4.5 | 394 | Vegetable | FFQ | GC | Cancer registry | 0.71 servings per 1000 calories/d  1.15  1.56  2.08  3.15 | 1  1.22 (0.91-1.65)  1.28 (0.94-1.73)  1.04 (0.94-1.73)  0.96 (0.68-1.37) | Age, BMI, total energy, education, alcohol intake, cigarette-smoke-dose, usual activity throughout the day, vigorous physical activity, ethnicity, and fruit and vegetable intake when appropriate |
|  |  |  |  |  |  | Fruit |  |  |  | 0.45 servings per 1000 calories/d  0.98  1.46  2.06  3.20 | 1  1.10 (0.53-1.03)  0.74 (0.53-1.03)  0.91 (0.66-1.27)  1.04 (0.75-1.45) |  |
|  |  |  |  |  |  | Total vegetable and fruit |  |  |  | 1.51 servings per 1000 calories/d  2.41  3.18  4.11  5.81 | 1  1.01 (0.73–1.39)  0.91 (0.64–1.29)  1.11 (0.79–1.58)  0.86 (0.58–1.29) |  |
| Chyou et al. 1990 | US | 57 | M: 472 | 18 | 111 | Vegetable | Dietary recall | GC | Cancer registry | 0 g/d  0-40  40-80  ≥80 | 1  1.20 (0.80-2.00)  0.90 (0.50-1.50)  0.70 (0.40-1.10) | Age, smoking |
| Tran et al. 2005 | China | 40-69 | M/F: 29,584 | 15 | 1452 | Vegetable | FFQ | GCC | Cancer registry | ≤549 times/year  549–732  732–915  >915 | 1  0.94 (0.80-1.10)  1.03 (0.88-1.20)  1.17 (0.96-1.42) | Age, sex |
|  |  |  |  |  |  |  |  | GNCC |  | ≤549 times/year  549–732  732–915  >915 | 1  1.30 (0.99-1.71)  1.43 (1.09-1.87)  1.04 (0.71-1.53) |  |
|  |  |  |  |  |  | Fruit |  | GCC |  | ≤1 times/year  1–5  5–13  >13 | 1  1.02 (0.86-1.20)  0.84 (0.71-1.00)  0.89 (0.75-1.05) |  |
|  |  |  |  |  |  |  |  | GNCC |  | ≤1 times/year  1–5  5–13  >13 | 1  0.99 (0.73-1.33)  1.14 (0.86-1.51)  0.95 (0.71-1.28) |  |
| Ko et al. 2013 | Korea | 30-90 | M/F: 9,724 | 8.5 | 152 | Vegetable | FFQ | GC | Cancer registry | Almost never  1–4 times/month 1–4 times/week  ≥1 time/day | 1  0.80 (0.28-2.29)  0.66 (0.25-1.70)  0.68 (0.27-1.68) | Age, sex, cigarette smoking, BMI, alcohol drinking, and area of residence. |
|  |  |  |  |  |  | Fruit |  |  |  | Almost never  1–4 times/month 1–4 times/week  ≥1 time/day | 1  1.12 (0.58-2.18)  1.42 (0.75-2.68)  1.10 (0.55-2.22) |  |
| Nomura et al. 1990 | US | 46-65 | M: 7,990 | 10.6 | 150 | Fruit | FFQ | GC | Cancer registry | ≤1 times/week  2-4  ≥5 | 1  1.00 (0.60-1.70)  0.80 (0.50-1.30) | Age |
| Inoue et al. 1996 | Japan | >30 | M/F: 1041 | 6 | 69 | Fruit | FFQ | GC | Cancer registry | Rare  Daily | 1  1.04 (0.78-1.39) | Age |
| Li et al. 2010 | Japan | 40-79 | M/F: 42,470 | 9 | 806 | Citrus fruits | FFQ | GC | Cancer registry | <2 times/week  3–4 times/week Daily | 1  0.99 (0.83–1.19)  0.99 (0.80–1.21) | Age, sex, job status, years of education, BMI, time engaging in sports or exercise, time spent walking, cigarette smoking, alcohol drinking, history of hypertension, diabetes mellitus and gastric ulcer, family history of cancer, daily total energy intake, consumption of rice, daily consumption of miso soup, daily consumption of soybean products, total meat, total fish, dairy products, other fruits, total vegetables and consumption of oolong tea, black tea, coffee and green tea |
| Botterweck et al. 1998 | Netherlands | 55-59 | M/F: 3,405 | 6.3 | 264 | Total vegetable and fruit | FFQ | GC | Cancer registry | 190 g/d  276  345  418  544 | 1  0.74 (0.50-1.09)  0.69 (0.47-1.03)  0.81 (0.55-1.20)  0.72 (0.48-1.10) | Age, smoking, smoking, education, stomach disorders, and family history of stomach cancer |
| Galanis et al. 1998 | US | >18 | M/F: 11,907 | 14.8 | 108 | Fruit | FFQ | GC | Cancer registry | 0-6 times/week  ≥7 rimes/week | 1  0.60 (0.40-0.90) | Age, years of education, Japanese place of birth, and gender. Analyses among men were also adjusted for  cigarette smoking and alcohol intake status. |
| Terry et al. 1998 | Sweden | 36-75 | M/F: 11546 | 21 | 116 | Total vegetable and fruit | Questionnaire | GC | Cancer registry | High  Moderate  Small  None/very little | 1  2.07 (1.09-3.92)  1.91 (0.95-3.88)  5.53 (1.67-18.31) | Age, gender, alcohol intake, smoking, BMI at age 25 years and childhood socio-economic status. |
| Wang et al. 2017 | China, Japan and Korea | 59.5 | M/F: 1970 | 6.2 | 810 | Vegetable | FFQ | GNCC | Cancer registry | NR | 1  0.83 (0.64–1.09)  0.76 (0.57–1.01)  0.94 (0.70–1.26) | Age, sex, date of biological collection, smoking, total energy intake, Omp and HP 0305 status (Omp and/or HP 0305 negative, Omp and HP 0305 positive), and fruit intake or vegetable intake (whichever one is not the main exposure). |
|  |  |  |  |  |  | Fruit |  |  |  | NR | 1  0.85 (0.66–1.10)  0.78 (0.59–1.03)  0.65 (0.48–0.88) |  |

Abbreviation: RR: relative risk- CI: confidence interval- M: male- F: female- FFQ: food frequency questionnaire- US: United States- BMI: body mass index- GC: gastric cancer- GCC: gastric cardia cancer- GNCC: gastric noncardia cancer-NR: not-reported

**Supplemental Table 3**: Quality assessment of prospective cohort studies included in the current systematic review and meta-analysis on the association between fruit and vegetable intake and risk of gastric cancer based on the Newcastle-Ottawa scale

| Author | Representativeness of the exposed cohort | Selection of the non-exposed cohort | Ascertainment of exposure | Outcome of interest was not present at the start of the study | Energy adjustment | Controls for any additional factor | Assessment of outcome | Follow-up long enough | Adequacy of follow-up of cohorts | Total |
| --- | --- | --- | --- | --- | --- | --- | --- | --- | --- | --- |
| Steevens et al. 2011 | * | * |  | * |  | * | * | * | * | 7 |
| Shimazu et al. 2014 | * | * |  | * | * | * | * | * | * | 8 |
| Nouraie et al. 2005 | * | * |  | * | * | * | * | * | * | 8 |
| Larsson et al. 2006 | * | * |  | * | * | * | * |  | * | 7 |
| Gonzalez et al. 2012 | * | * |  | * | * | * | * | * | * | 8 |
| Epplein et al.2010 | * | * |  | * | * | * | * |  | * | 7 |
| Freedman et al. 2008 | * | * |  | * | * | * | * |  | * | 7 |
| Chyou et al. 1990 |  | * | * | * |  | * | * | * | * | 7 |
| Tran et al. 2005 | * | * | * | * |  | * | * | * | * | 8 |
| Ko et al. 2013 | * | * |  | * |  | * | * |  | * | 6 |
| Nomura et al. 1990 | * | * |  | * |  | * | * | * | * | 7 |
| Inoue et al. 1996 | * | * |  | * |  | * | * |  | * | 7 |
| Li et al. 2010 | * | * |  | * | * | * | * |  | * | 7 |
| Botterweck et al. 1998 | * | * |  | * |  | * | * |  | * | 6 |
| Galanis et al. 1998 | * | * |  | * |  | * | * | * | * | 7 |
| Terry et al. 1998 | * | * |  | * |  | * | * | * | * | 7 |
| Wang et al. 2017 | * | * |  | * | * | * | * |  | * | 7 |

**Supplemental Table 4:** Stratified analysis on association between fruit and vegetable intake and risk of gastric cancer in adults aged ≥18 years

|  | | | ***n*^2^** | **Pooled ES (95% CI)^3^** | **I^2^ (%)^4^** | **P-heterogeneity^5^** |
| --- | --- | --- | --- | --- | --- | --- |
| **Vegetable** | | | | | | |
|  | Location | |  |  |  |  |
|  |  | US | 2 | 0.87 (0.65-1.16) | 1.2 | 0.31 |
|  |  | Non-US | 9 | 0.91 (0.81-1.03) | 27.9 | 0.19 |
|  | Sex | |  |  |  |  |
|  |  | Male and female | 8 | 0.91 (0.80-1.04) | 36.5 | 0.13 |
|  |  | Male | 2 | 0.83 (0.58-1.20) | 0 | 0.33 |
|  |  | Female | 1 | 0.89 (0.60-1.32) | - | - |
|  | Follow-up duration | |  |  |  |  |
|  |  | >10 years | 6 | 0.93 (0.81-1.06) | 36.6 | 0.16 |
|  |  | <10 years | 5 | 0.85 (0.69-1.04) | 0 | 0.44 |
|  | Dietary assessment tools | |  |  |  |  |
|  |  | FFQ | 10 | 0.92 (0.83-1.02) | 19.3 | 0.26 |
|  |  | Food record | 1 | 0.70 (0.42-1.16) | - | - |
|  | Adjustment for energy | |  |  |  |  |
|  |  | Yes | 7 | 0.87 (0.79-0.96) | 0 | 0.73 |
|  |  | No | 4 | 0.94 (0.75-1.20) | 43.6 | 0.15 |
|  | Adjustment for BMI | |  |  |  |  |
|  |  | Yes | 3 | 0.91 (0.73-1.13) | 0 | 0.78 |
|  |  | No | 8 | 0.90 (0.78-1.03) | 40.6 | 0.10 |
|  | Cancer subsite | |  |  |  |  |
|  |  | Gastric cardia cancers | 5 | 1.08 (0.92-1.27) | 0 | 0.60 |
|  |  | Gastric non-cardia cancers | 8 | 0.88 (0.78-0.99) | 0 | 0.94 |
| **Fruit** | | | | | | |
|  | Location | |  |  |  |  |
|  |  | US | 3 | 0.81 (0.58-1.13) | 53.3 | 0.11 |
|  |  | Non-US | 10 | 0.87 (0.80-0.95) | 0 | 0.96 |
|  | Sex | |  |  |  |  |
|  |  | Male and female | 10 | 0.87 (0.80-0.96) | 0 | 0.64 |
|  |  | Male | 2 | 0.80 (0.56-1.12) | 0 | 0.97 |
|  |  | Female | 1 | 0.98 (0.65-1.49) | - | - |
|  | Follow-up duration | |  |  |  |  |
|  |  | >10 years | 7 | 0.86 (0.78-0.94) | 0 | 0.62 |
|  |  | <10 years | 6 | 0.93 (0.77-1.13) | 0 | 0.77 |
|  | Dietary assessment tools | |  |  |  |  |
|  |  | FFQ | 13 | 0.87 (0.80-0.94) | 0 | 0.82 |
|  |  | Food record | 0 | - | - | - |
|  | Adjustment for energy | |  |  |  |  |
|  |  | Yes | 7 | 0.88 (0.79-0.99) | 0 | 0.86 |
|  |  | No | 6 | 0.85 (0.76-0.96) | 0 | 0.43 |
|  | Adjustment for BMI | |  |  |  |  |
|  |  | Yes | 3 | 0.93 (0.76-1.15) | 0 | 0.56 |
|  |  | No | 10 | 0.86 (0.78-0.94) | 0 | 0.76 |
|  | Cancer subsite | |  |  |  |  |
|  |  | Gastric cardia cancers | 5 | 0.88 (0.76-1.02) | 0 | 0.99 |
|  |  | Gastric non-cardia cancers | 8 | 0.87 (0.77-0.98) | 0 | 0.68 |
| **Fruit and vegetable** | | | | | | |
|  | Location | |  |  |  |  |
|  |  | US | 1 | 0.91 (0.64-1.30) | - | - |
|  |  | Non-US | 5 | 0.70 (0.54-0.92) | 63.2 | 0.02 |
|  | Sex | |  |  |  |  |
|  |  | Male and female | 6 | 0.75 (0.61-0.93) | 55.2 | 0.04 |
|  |  | Male | 0 | - | - | - |
|  |  | Female | 0 | - | - | - |
|  | Follow-up duration | |  |  |  |  |
|  |  | >10 years | 3 | 0.72 (0.49-1.06) | 74 | 0.02 |
|  |  | <10 years | 3 | 0.74 (0.56-0.98) | 25.9 | 0.26 |
|  | Dietary assessment tools | |  |  |  |  |
|  |  | FFQ | 6 | 0.75 (0.61-0.93) | 55.2 | 0.04 |
|  |  | Food record | 0 | - | - | - |
|  | Adjustment for energy | |  |  |  |  |
|  |  | Yes | 3 | 0.76 (0.60-0.98) | 23.8 | 0.27 |
|  |  | No | 3 | 0.66 (0.40-1.08) | 74.7 | 0.01 |
|  | Adjustment for BMI | |  |  |  |  |
|  |  | Yes | 3 | 0.67 (0.41-1.11) | 71.3 | 0.03 |
|  |  | No | 3 | 0.77 (0.59-0.99) | 47.3 | 0.15 |
|  | Cancer subsite | |  |  |  |  |
|  |  | Gastric cardia cancers | 2 | 0.83 (0.56-1.23) | 0 | 0.69 |
|  |  | Gastric non-cardia cancers | 3 | 0.84 (0.70-1.01) | 0 | 0.59 |
| **Citrus fruit** | | |  |  |  |  |
|  | Location | |  |  |  |  |
|  |  | US | 1 | 1.09 (0.85-1.39) | - | - |
|  |  | Non-US | 5 | 0.85 (0.73-0.99) | 25.3 | 0.25 |
|  | Sex | |  |  |  |  |
|  |  | Male and female | 4 | 0.90 (0.75-1.09) | 56.8 | 0.07 |
|  |  | Male | 1 | 0.70 (0.41-1.18) | - | - |
|  |  | Female | 1 | 0.94 (0.62-1.42) | - | - |
|  | Follow-up duration | |  |  |  |  |
|  |  | >10 years | 2 | 0.77 (0.59-1.01) | 46.3 | 0.17 |
|  |  | <10 years | 4 | 0.99 (0.86-1.14) | 0 | 0.51 |
|  | Dietary assessment tools | |  |  |  |  |
|  |  | FFQ | 6 | 0.90 (0.77-1.04) | 37.2 | 0.15 |
|  |  | Food record | 0 | - | - | - |
|  | Adjustment for energy | |  |  |  |  |
|  |  | Yes | 5 | 0.96 (0.85-1.09) | 0 | 0.54 |
|  |  | No | 1 | 0.66 (0.48-0.89) | - | - |
|  | Adjustment for BMI | |  |  |  |  |
|  |  | Yes | 3 | 0.98 (0.86-1.12) | 0 | 0.44 |
|  |  | No | 3 | 0.74 (0.59-0.93) | 0 | 0.39 |
|  | Cancer subsite | |  |  |  |  |
|  |  | Gastric cardia cancers | 3 | 0.62 (0.39-0.99) | 66.9 | 0.04 |
|  |  | Gastric non-cardia cancers | 5 | 1.01 (0.79-1.28) | 47.2 | 0.10 |

^1^Abbreviations: BMI, body mass index; CI, confidence interval; ES, effect size; FFQ, food frequency questionnaire; US, United States

^2^Number of effect sizes

^3^Obtained from the random-effects model

^4^Inconsistency- the percentage of variation across studies due to heterogeneity

^5^Obtained from the Q-test

**Online Supporting Material**

**Supplemental Figure 1**





Forest plot for the risk of gastric cancer based on 100 g/day increase in total vegetable intake in adults aged >18 years. Horizontal lines represent 95% CIs. Diamonds represent the pooled estimates from the random-effects analysis. RR: relative risk, CI: confidence interval

**Online Supporting Material**

**Supplemental Figure 2**





Forest plot for the risk of gastric cancer based on 100 g/day increase in total fruit intake in adults aged >18 years. Horizontal lines represent 95% CIs. Diamonds represent the pooled estimates from the random-effects analysis. RR: relative risk, CI: confidence interval

**Online Supporting Material**

**Supplemental Figure 3**





Forest plot for the risk of gastric cancer based on 200 g/day increase in total vegetable and fruit intake in adults aged >18 years. Horizontal lines represent 95% CIs. Diamonds represent the pooled estimates from the random-effects analysis. RR: relative risk, CI: confidence interval

**Online Supporting Material**

**Supplemental Figure 4**





Forest plot for the risk of gastric cancer based on 50 g/day increase in citrus fruit intake in adults aged >18 years. Horizontal lines represent 95% CIs. Diamonds represent the pooled estimates from the random-effects analysis. RR: relative risk, CI: confidence interval
